# Targeting muscle wasting: Bioinformatics-derived ubiquitination genes as potential therapeutic targets for sarcopenia

**DOI:** 10.1097/MD.0000000000047161

**Published:** 2026-01-16

**Authors:** XiaoMing Liu, Ren Li, YingYan Kuang

**Affiliations:** a Rehablitation Department, Shenzhen Bao’an Chinese Medicine Hospital, Guangzhou University of Chinese Medicine, Shenzhen, Guangdong, China.

**Keywords:** bioinformatics analysis, differentially expressed genes, sarcopenia, therapeutic targets, ubiquitin-proteasome system

## Abstract

Sarcopenia is a major health concern characterized by progressive loss of muscle mass and function among the elderly. Its prevalence ranges from 10% to 27% in individuals over 60 and increases further in those above 80. The condition reduces mobility and strength, increasing risks of falls, fractures, and other age-related health issues. The ubiquitin-proteasome system (UPS) plays a central role in muscle protein degradation and contribute to muscle wasting. However, therapeutic strategies targeting this pathway remain poorly understood. This study aimed to identify ubiquitination-related genes as potential therapeutic targets for sarcopenia using integrative bioinformatics analysis. This study utilized bioinformatics approaches to identify potential therapeutic targets for sarcopenia. Six GEO datasets (GSE136344, GSE28422, GSE1428, GSE8479, GSE9103, and GSE38718) were analyzed. Data were pre-processed, normalized, and batch effects were removed using the R packages “affy” and “sva.” Differentially expressed genes were identified using the “limma” package. Weighted gene co-expression network analysis was performed to identify disease-associated genes. Functional enrichment analyses were conducted using the “clusterProfiler” package for Gene Ontology (GO) is a standardized, structured vocabulary (ontology) for describing the functions of genes and gene products across all species and Kyoto Encyclopedia of Genes and Genomes (KEGG) is a comprehensive database resource that integrates genomic, chemical, and systems-level functional information pathways. Differentially ubiquitinated genes were identified by intersecting differentially expressed genes with ubiquitination-related genes from MSigDB. Protein–protein interaction networks were constructed using STRING, and machine learning algorithms were applied to screen for diagnostic signature genes. Drug-gene interactions were analyzed using DGIdb, and molecular docking was performed using AutoDock. A total 734 differentially expressed genes were identified, including 373 up-regulated and 361 down-regulated genes. Gene set enrichment analysis revealed significant enrichment in 3 KEGG pathways. Weighted gene co-expression network analysis identified 1016 disease-associated genes for functional enrichment. Thirteen differentially ubiquitinated genes were identified, and 6 key diagnostic signature genes (CBLB, PSMD6, RNF115, SMAD3, UCHL3, and ZBTB16) were selected through machine learning. Five genes (CBLB, RNF115, SMAD3, UCHL3, and ZBTB16) exhibited significant differences between older and younger groups, with ROC AUC values > 0.7. Drug prediction identified ASPARTIC ACID and CETYLPYRIDINIUM as potential agents targeting CBLB and SMAD3. This study identified key differentially ubiquitinated genes with potential therapeutic implications for sarcopenia. The findings highlight the importance of the UPS in muscle wasting and provide a foundation for further mechanistic and therapeutic research.

## 1. Introduction

As a common progressive skeletal muscle disorder in the clinical setting, sarcopenia may present with skeletal muscle mass loss and dysfunction in the sufferers.^[1]^ Its prevalence is calculated to be 10%–27% among individuals aged 60 years and older,^[2]^ even higher in those aged 80 years and older. The consequences of sarcopenia extend beyond physical health, profoundly impacting the elderly population. It contributes to a noticeable reduction in mobility and strength, elevating the likelihood of serious incidents such as falls and bone fractures. This condition may also frequently coexist with other age-related health issues, such as cardiac diseases, diabetes, and bone density loss, further worsening the overall well-being and daily functioning of older adults.

Sacrcopenia is a complex disorder that develops through several biological processes including impaired protein synthesis, mitochondrial dysfunction, chronic inflammation, hormonal changes, and increased protein degradation.^[3,4]^ Among these mechanisms, the ubiquitin proteasome system (UPS) plays an important role in the breakdown of muscle proteins. In this system, target proteins are labeled with ubiquitin and then degraded by proteasome, leading to accelerated muscle protein loss.^[5,6]^ This process is particularly correlated with aging related muscle atrophy where enhanced proteolysis involves the progressive loss of muscle mass and function. Recent studies have demonstrated that UPS plays as a critical regulator of skeletal muscle homeostasis and degradation,^[7]^ making it a promising therapeutic target for mitigating muscle loss and restoring muscle function in sarcopenia.

Although the role of UPS has been recognized in muscle wasting, by far there is no effective therapy to prevent or reverse sarcopenia. Current strategies such as exercise and nutritional supplementation can offer some benefits but often brought modest improvements, particularly in advanced stages of this disorder.^[8,9]^ Notably, the specific ubiquitin related genes and molecules which might serve as diagnostic markers or therapeutic targets still remain poorly understood, largely limiting the development of precise molecular interventions for sarcopenia treatment.

To bridge this knowledge gap, here we employed an integrative bioinformatics framework to systematically identify ubiquitination related genes associated with sarcopenia and examine their potential as therapeutic targets. Through the multiple gene expression datasets, weighted gene co-expression network analysis (WGCNA), functional enrichment analysis and machine learning, we aimed to explore the key UPS associated genes and predict potential compounds that might regulate their function.

## 2. Materials and methods

### 2.1. Data sources and pre-processing

Eligible datasets used for analysis in this study were sourced online from NCBI GEO database.^[10]^ The inclusion criteria were as follows: sarcopenia-independent expression profiles; datasets containing older adults with sarcopenia and control young adults, and test specimens from datasets derived from human tissues. Table 1 summarizes the information on relevant datasets.

**Table 1:** Sarcopenia GEO dataset.

	Accession ID	Old	Young	Platform
Training set	GSE136344	12	11	GPL5175
	GSE28422	12	15	GPL570
	GSE1428	12	10	GPL96
Validation set	GSE8479	25	26	GPL2700
	GSE9103	10	10	GPL570
	GSE38718	8	14	GPL570

Training set: By downloading the data “GSE136344_series_matrix.txt.gz,” GSE136344 has 12 older samples (Old) and 11 younger samples (Young). By downloading the data “GSE28422_series_matrix.txt.gz,” GSE28422 has 12 older samples (Old) and 15 younger samples (Young). In addition, by downloading the data “GSE1428_series_matrix.txt.gz,” GSE1428 has 12 older samples (Old) and 10 younger samples (Young).

Validation set: By downloading the data “GSE8479_RAW.tar,” GSE8479 has 25 old (Old) and 26 young (Young) samples. By downloading the raw data “GSE9103_RAW.tar,” GSE9103 has 10 old samples (Old) and 10 young samples (Young). Then, by downloading the raw data “GSE38718_RAW.tar,” GSE38718 has 8 aged samples (Old) and 14 control samples (Young).

The raw data were processed using the R package “affy” (Version 1.74.0,), and the expression matrices were background corrected and normalized. The gene annotation was performed by downloading the mRNA probe expression matrix files corresponding to each dataset and the annotation files of the corresponding sequencing platforms. The probes were converted into gene symbols one by one, with the removal of unqualified probes that did not match the gene symbols. The average value of different probes mapped to the same gene was taken as the expression value of the gene, which was then obtained as the expression matrix for the subsequent analysis. The gene expression matrix was obtained for subsequent analysis. Then, R package “sva” (Version 3.50.0)^.[11]^ was used to analyze GSE136344, GSE28422 and GSE1428, Then, the package was used to remove and merge the batch effects on the expression profiles of the training and validation sets for the subsequent analysis.

Given that the individual datasets analyzed in this study included relatively modest sample sizes (10–25 samples per group), we considered the potential impact on statistical power. To reduce these limitations, we integrated multiple independent cohorts and applied rigorous normalization, differential expression analysis, and multiple-testing corrections using a false discovery rate (FDR) threshold of 0.05. Although small sample sizes may reduce power to detect subtle differences, the use of multiple datasets and robust analytical strategies enhanced the reliability of the identified candidate genes. Therefore, these results should be interpreted as exploratory and hypothesis-generating, with future studies in larger cohorts needed to confirm these findings.

### 2.2. Acquisition of differentially expressed genes (DEGs)

The R package “limma” (Version 3.52.4,) was used to analyze the differences between the Old versus Young comparison groups in the training set.^[12]^ By employing difference analysis, this study calculated corresponding *P* values and log2FC of the genes. We evaluated the genes at 2 levels, multiplicity of difference and significance. The threshold of |log2FC| ≥ 0.263 (corresponding to |FC| ≥ 1.2) and adjusted *P*-value < .05 was adopted based on previously published transcriptomic studies employing similar cutoffs for moderate effect sizes.^[13]^ For all differential expression analyses, functional enrichment analyses (GO and KEGG), and gene set enrichment analyses (GSEA), multiple testing correction was performed using the Benjamini–Hochberg FDR method, and adjusted *P*-values (FDR values) were consistently applied to determine statistical significance.

### 2.3. GSEA analysis

Through the analysis of the gene set (KEGG) by GSEA,^[14]^ this study intended to analyze whether there was a significant difference between the gene set in the Old vs. Young comparison groups based on the training set data. Gene sets with adjust. *P* < .05 were selected for presentation.

### 2.4. Identification of disease-associated genes by the weighted gene co-expression network analysis (WGCNA)

The WGCNA method was utilized to screen for and identify disease-associated genes. Firstly, we assumed that the gene network obeyed a scale-free network, and defined the gene co-expression correlation matrix, the neighbor-joining function formed by the gene network, then calculated the coefficients of dissimilarity of different nodes, and identified the modules of the gene collection that were associated with the phenotypes. The R package “WGCNA”^[15]^ (Version 1.71) was used to to analyze the matrix data of DEGs to compare between the Old vs. Young comparison groups as phenotypic traits. The absolute median difference in gene expression was ranked from largest to smallest, and the genes ranked as the top-7000 were selected for the expression matrix. The absolute value of module correlation > 0.45 and *P* < .05 was used as the threshold to filter all genes in the relevant module for subsequent analysis.

### 2.5. Functional enrichment analyses

For the modular genes obtained in the previous step, the R package “clusterProfiler” (Version 4.10.0)^[16]^ for enrichment analysis to explore the entries of functional pathways involved in key genes. Gene Ontology, or GO for short can realize comprehensive description of genes and gene products in organisms by providing a set of dynamically updated controlled vocabulary. GO describes the molecular function, cellular component, and biological process of genes, respectively. Benjamini & Hochberg method was used for multiple test correction to obtain the corrected (i.e. adjust) *P* value (threshold, 0.05). Finally, the most significant TOP10 GO enrichment results were selected to be displayed along with the TOP30 KEGG enrichment results.

### 2.6. Identification of differentially ubiquitinated genes in sarcopenia

Using the MSigDB,^[17]^ the data from one GO genome (GO UBIQUITIN DEPENDENT ERAD PATHWAY), one KEGG genome (KEGG UBIQUITIN MEDIATED PROTEOLYSIS), one KEGG genome (KEG UBIQUITIN MEDIATED) and three REACTOME genomes (REACTOME ANTIGEN PROCESSING UBIQUITINATION PROTEASOME DEGRADATION, REACTOME DEUBIQUITINATION, and REACTOME PROTEIN UBIQUITINATION) were obtained from a total of 674 ubiquitination-related genes (URGs).^[18,19]^

An intersection was taken from the differentially up- and down-regulated genes obtained from the limma screen as well as all the genes within the module selected by WGCNA to meet the screening criteria. Then, all the intersected genes were taken to be intersected with the URGs to obtain corresponding genes as the sarcopenia-associated differentially ubiquitinated genes for the subsequent analyses.

### 2.7. Protein interaction network construction

The interactions between gene product proteins of sarcopenia URGs were clarified by visiting STRING (Version 12.0)^.[20]^ The construction of PPI network was completed based on a threshold of combined_score > 0.15.

### 2.8. Machine learning (ML) screening for diagnostic signature genes

Three ML algorithms, Least Absolute Shrinkage and Selection Operator (LASSO) is a regression method that performs both variable selection and regularization via an L1-penalty, resulting in sparse coefficient estimates, Random Forest (RF) and SVM-RFE, were utilized to further screen the list of potential feature genes for sarcopenia diagnosis. LASSO analyses used regularized penalty parameters to select feature variables through 10-fold cross-validation. SVM- Recursive Feature Elimination (RFE) in the RFE algorithm (a supervised ML method) ranks feature genes associated with sarcopenia. Predictive performance was assessed by 10-fold cross-validation to identify feature genes. RF outperformed linear discriminant analysis and mean square error methods in selecting relevant features and removing redundant features. Feature selection was performed by ten-fold cross validation, and genes with relative importance MeanDecreaseGini > 2.5 were selected as significant variables. LASSO regression, SVM-RFE, and RF analyses were performed using the R package “glmnet (version 4.1-6),”^[21]^ “e1071” (version 1.7-14)^[22]^ and “RandomForest” (version 4.7-1.1).^[23]^ The intersected genes were considered as key diagnostic signature genes in sarcopenia.

### 2.9. Validation and performance evaluation of characterized genes

Differences of the characterized genes among different states of sarcopenia were compared by Wilcoxon test, and the working characteristics of the subjects were plotted in all datasets (training and validation sets) by utilizing pROC^[24]^ (Version1.18.5) in the R package. ROC curves and area under ROC curve (AUC) were employed to assess the accuracy of the diagnosis of the characterized genes. Diagnostic genes with *P* < .05 and AUC > 0.7 were retained for subsequent analysis.

### 2.10. Drug forecasting

DGIdb contains > 10,000 genes and 20,000 drugs covering nearly 70,000 drug-gene interactions.^[25]^ DGIdb was employed here to analyze the relationship between the characterized genes and drugs, and the input genes were selected as drugs with interaction score > 0.2 and regulatory approval as “Approved.” Furthermore, molecular docking of the interactions was performed using AutoDock software. Firstly, the crystal structures of the characterized gene proteins were downloaded from PDB,^[26]^ and the water molecules and original ligands in the characterized gene proteins were removed by Pymol. After that, the processed proteins were imported into AutoDock Tools to perform hydrogenation, charge calculations, and nonpolar hydrogen atoms combinations. The appropriate Grid-Box size and genetic algorithm parameters were set in AutoDock Vina. Box size and genetic algorithm parameters in AutoDock Vina were utilized to run molecular docking. Finally, result visualization adopted Discovery Studio 2019 software.^[27]^ Previously obtained 3D structures of drugs and characterized genes were obtained from Pubchem and PDB.^[28]^

### 2.11. GESA analysis of molecular mechanisms of characterized genes

GSEA is a computational method for assessing whether a set of genes shows a statistically significant difference between 2 biological states. It was utilized here to analyze KEGG enrichment for the involvement of characterized genes. Adjusted *P* (FDR) < .05 and NES absolute value >1 were set as cutoff threshold criteria.

## 3. Results

### 3.1. Data de-batching and consolidation

**Figure 1.**
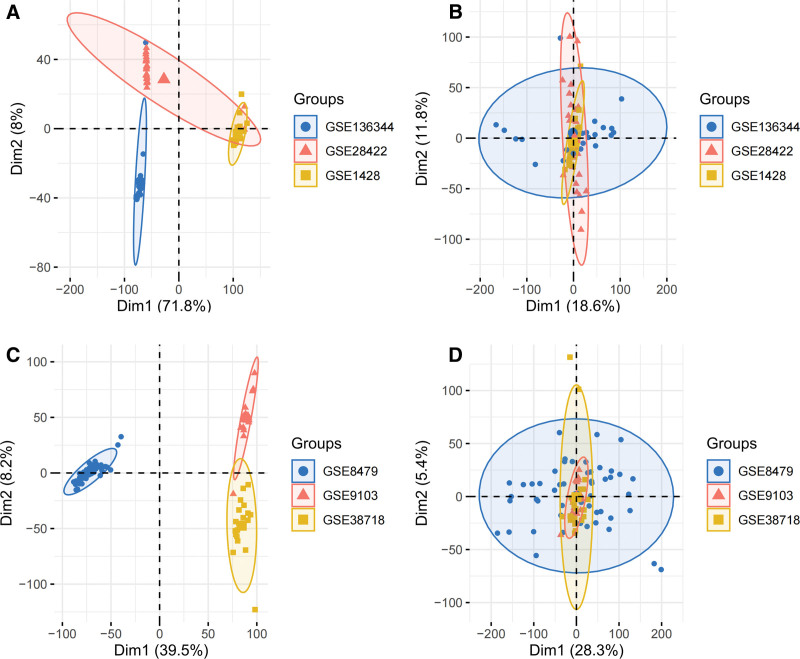
Surrogate Variable Analysis (SVA) is a computational and statistical framework designed to identify, estimate, and adjust for unobserved or unmodeled confounding factors in high-throughput genomic data (e.g., gene expression microarray or RNA-seq datasets) removal of batch effects:training set before (A) and after (B) removal of batch effects, validation set before (C) and after (D) removal of batch effects Expression data analysis PCA plots. PCA = Principal Component Analysis.

As described in the methodology, GSE136344, GSE28422, and GSE1428 were de-batched and combined to get 36 Old and 36 Young samples. Results were presented before and after data batching using Principal Component Analysis (Fig. 1A–B).

Simultaneously, de-batching of GSE8479, GSE9103, and GSE38718 was performed with 43 Old and 50 Young samples after merging. Similarly, results were also presented using Principal Component Analysis (Fig. 1C–D).

### 3.2. Identification of DEGs

**Figure 2.**
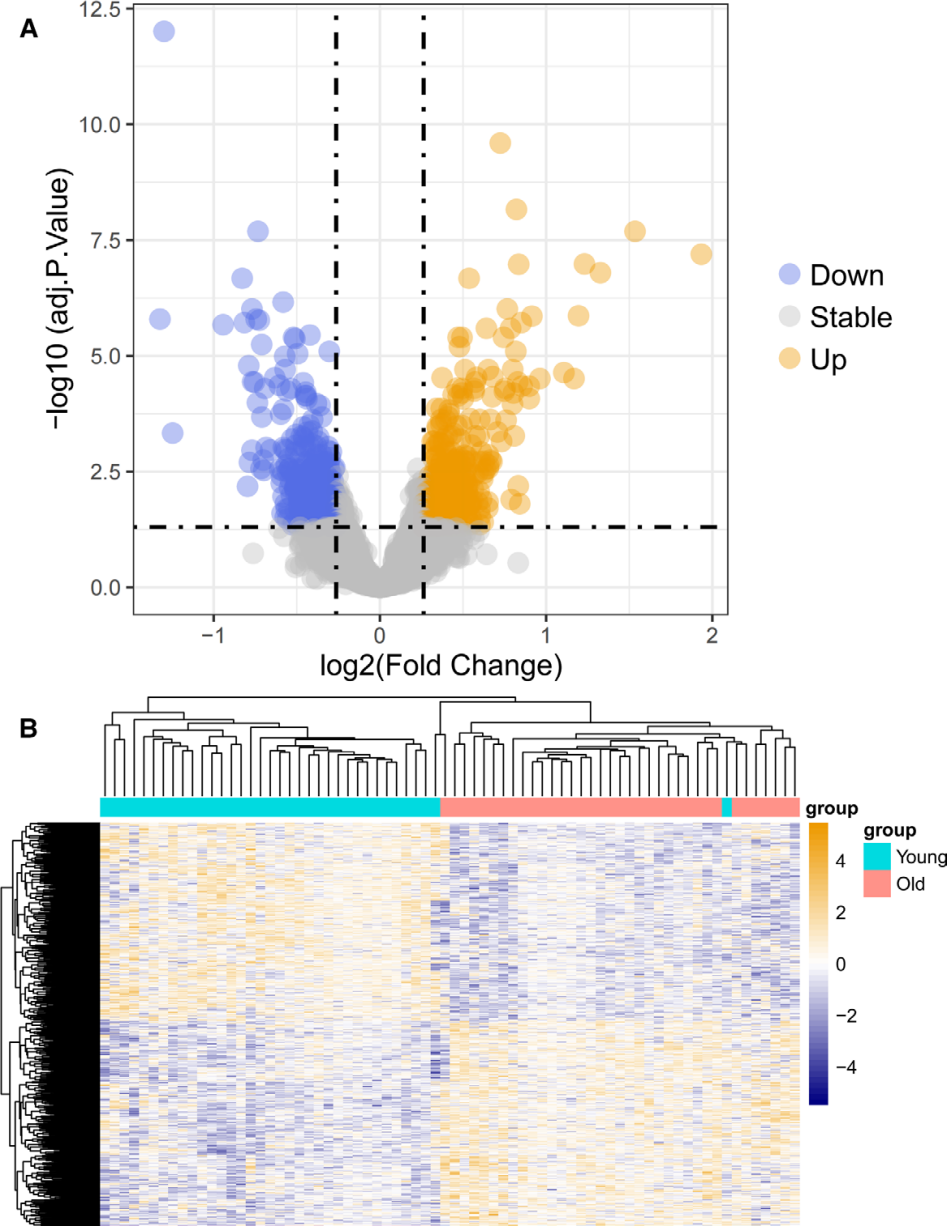
(A) Volcano plot of differentially expressed genes, with blue representing down-regulated genes and yellow representing up-regulated genes. (B) Heat map of differential gene expression.

Differential analytical results of Old vs Young are shown in Figure 2A. As detailed in deg_all.txt shown, 734 DEGs were obtained. Of these, 373 were up-regulated, and 361 were down-regulated genes. Figure 2B displays the heatmap of the identified genes.

### 3.3. GSEA analysis

**Figure 3.**
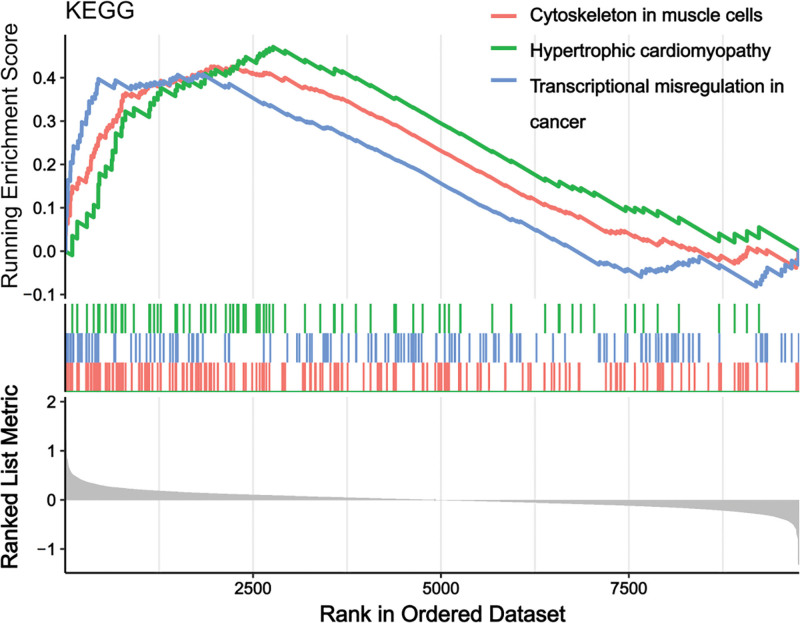
GSEA enrichment analysis results. GSEA = gene set enrichment analyses.

The gene set (KEGG) was analyzed and results of GSEA are shown in Figure 3 (KEGG_gseresult.csv), which significantly enriched to 3 significant pathways.

### 3.4. WGCNA identifies disease-associated genes

**Figure 4.**
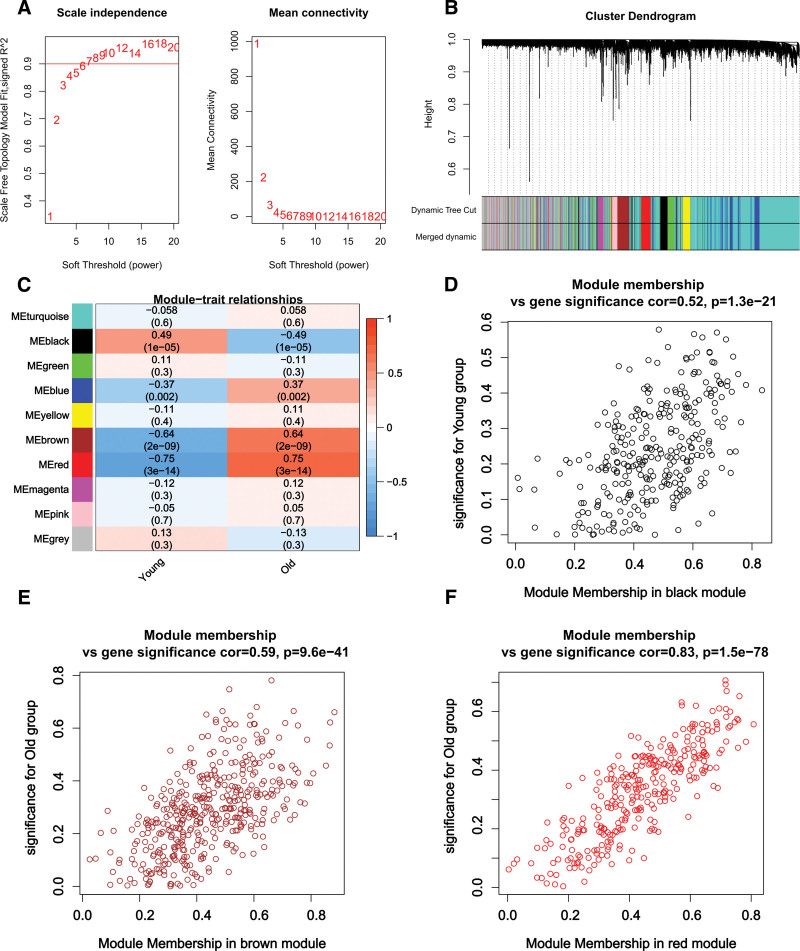
(A) Left: Plot of the choice of weight parameter power for the adjacency matrix. The horizontal axis represents the weight parameter power and the vertical axis represents the square of the correlation coefficient between log(k) and log(p(k)) in the corresponding network. Higher values of the squared correlation coefficient indicate that the network approximates the network-free scale distribution. The red line indicates the standard line where the squared value of the correlation coefficient reaches 0.9. Right: schematic diagram of the average connectivity of genes under different neighbor matrix weight parameters power parameter, the red line indicates the value of the average connectivity of the network nodes under the value of the neighbor matrix weight parameter power parameter taken in Figure A. (B) Tree diagram of the module division. Each color indicates a different module. (C) Heatmap of the correlation between each module and phenotype. (D) Correlation between module membership (x-axis) and gene significance (y-axis); Scatterplot of black modules versus phenotypic traits. (E) Scatterplot of brown modules versus phenotypic traits. (F) Scatterplot of red modules versus phenotypic traits.

Based on the expression data of DEGs, WGCNA analysis was performed with Old and Young as phenotypic traits. There was a need for exploring the value of neighbor-joining matrix weight parameter power to try to satisfy the scale-free network distribution prerequisites. It was completed by setting the range of choices for the network construction parameters and calculating the scale-free distribution topology matrix. In Figure 4A, we selected the value of power when the squared value of correlation coefficient reached 0.9 for the first time (red line), i.e., power = 7. Through clustering and dynamic pruning, modules were clustered involving the highly correlated genes. In addition to the gray module, 9 modules were classified based on the 7000 genes (Fig. 4B). This analysis further determined the correlation between each module and phenotype (Fig. 4C). Our subsequent concerns of analysis were brown (correlation:0.64, P:2e-09), red (correlation:0.64, P:3e-14), and black (correlation:0.49, P:1e-05) modules, followed by further correlation analysis of the 3 modules with the traits as shown in Figure 4D–F. Therefore, 1016 genes contained in the 3 modules were used as disease-associated genes for subsequent analysis.

### 3.5. GO and KEGG functional enrichment

**Figure 5.**
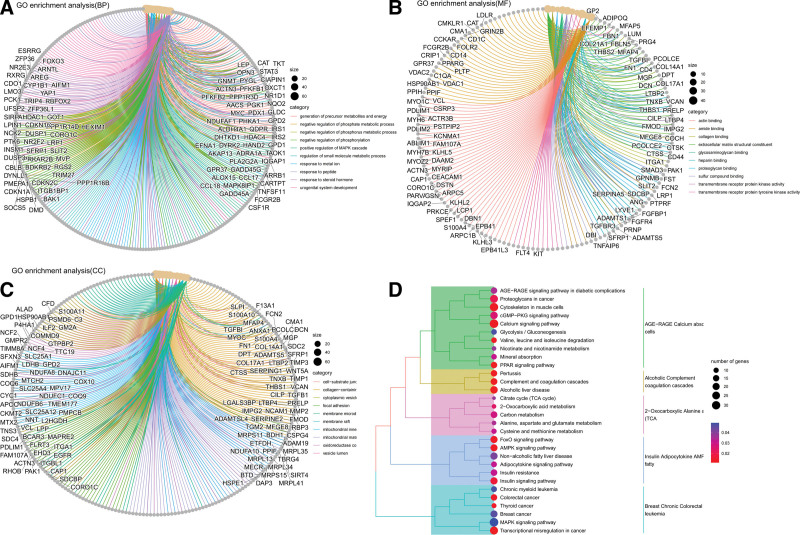
(A–C) Network diagram of GO enrichment analysis pathways. (D) Dendrogram of KEGG enrichment analysis.

Concerning the above 1016 modular genes to explore the functions of key genes involved in Term.Gene Ontology, the enrichment results are shown in Figure 5A–D, including GO analysis TOP10 results and KEGG analysis TOP30 results. TOP is sorted from smallest to largest in terms of *P* value (adjust *P* < .05) to obtain significantly enriched pathways.

### 3.6. Identification of differentially ubiquitinated genes in sarcopenia

**Figure 6.**
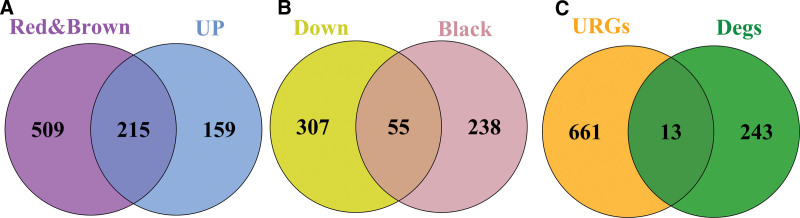
(A) Up-regulated genes vs WGCNA red module, all genes in brown module veen plot; (B) Down-regulated genes vs all genes in WGCNA black module veen plot. (C) Target differential genes vs ubiquitinated genes veen plot. WGC = weighted gene co-expression network analysis.

In view of the differential and WGCNA module genes, 215 up-regulated genes were identified along with 54 down-regulated genes (Fig. 6A–B). Subsequently, these genes were intersected with the database ubiquitination genes to take the intersection genes to get 13 differentially ubiquitinated genes (Fig. 6C). To minimize potential gene selection bias associated with intersecting WGCNA modules, DEGs, and URGs, we performed batch-effect correction and normalization across multiple datasets prior to analysis. The intersection strategy was applied to identify genes supported by multiple analytical approaches, thereby increasing confidence in their biological relevance and reducing false positives. Sensitivity checks did not reveal additional key genes outside the intersected set, suggesting that no major selection bias was introduced.

### 3.7. PPI network construction

**Figure 7.**
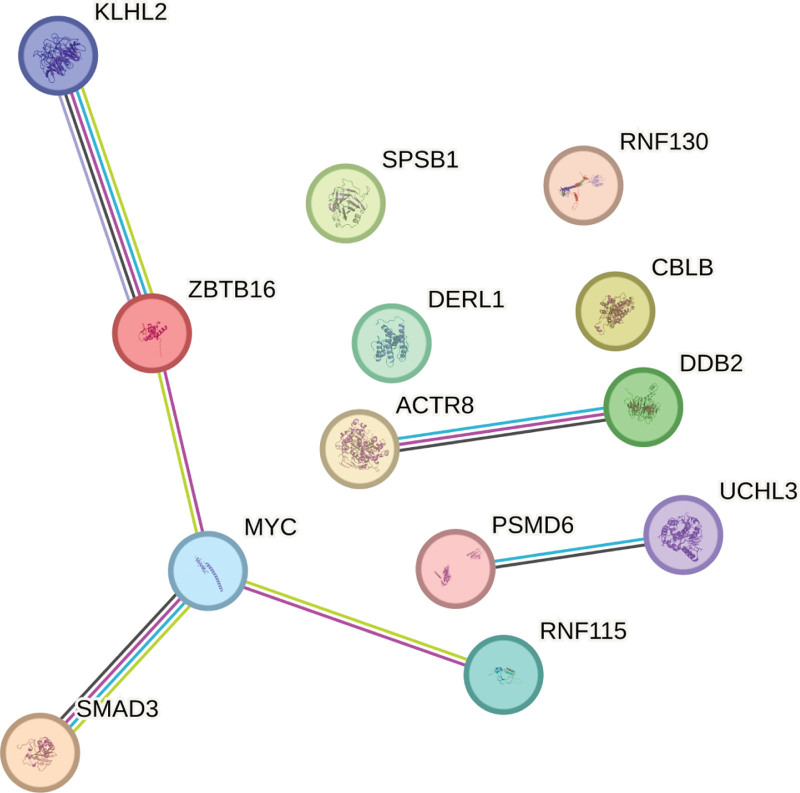
PPI network diagram.

The PPI network was constructed by STRING for the above differentially ubiquitinated genes, with the minimum interactions activity set to 0.4. The result file of PPI analysis was the attachment string_interactions_short.tsv, and the PPI network is shown in Figure 7. Among them, 13 genes were presented in the network.

### 3.8. ML algorithm-based feature gene selection

**Figure 8.**
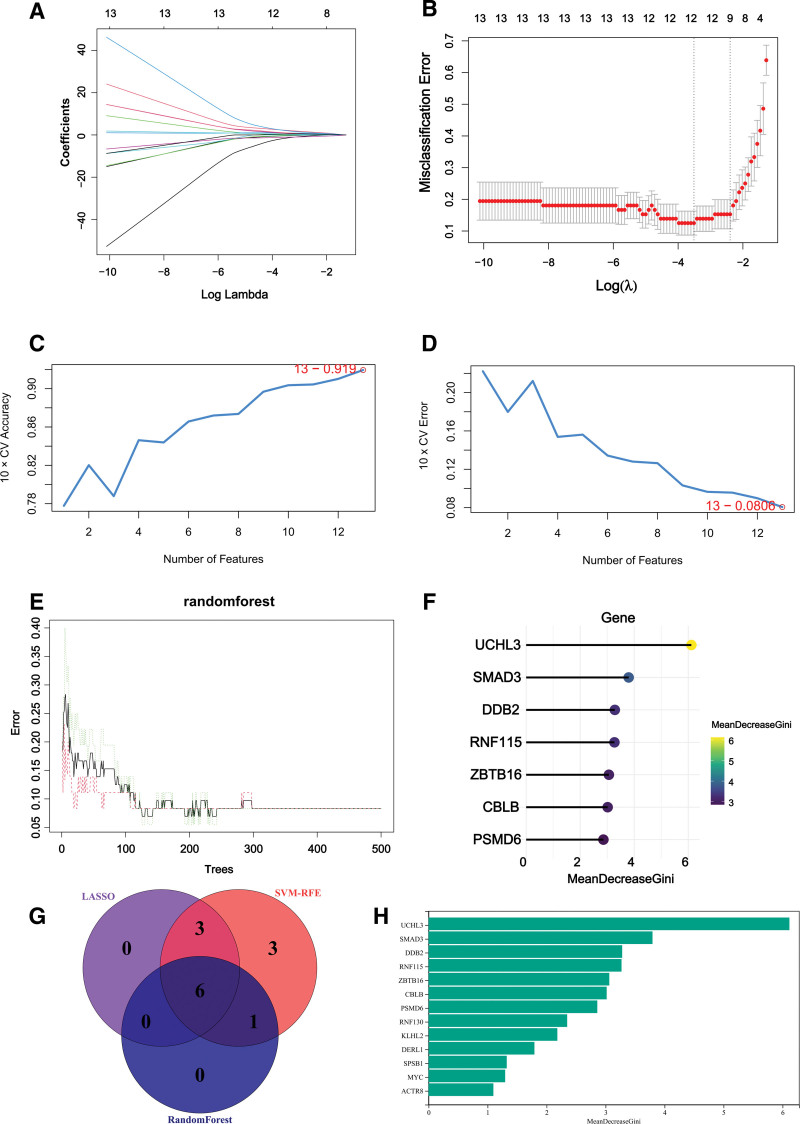
(A) Ten-fold cross-validation for optimized parameter selection in the LASSO model. Each curve corresponds to one gene. (B) LASSO coefficient profiles. The solid vertical line indicates the partial likelihood deviation SE. the right dashed line is drawn vertically at the optimal lambda. (C–D) SVM-RFE feature selection algorithm. (E) Random forests are used for the relationship between the number of trees and the error rate. (F) Arrangement of genes according to their relative importance. (G) Venn diagram showing feature genes shared by LASSO, Random Forest and SVM-RFE algorithms.

Three algorithms were applied to select feature genes among 13 key differentially ubiquitinated genes. For the LASSO algorithm, after ten times of cross-validation, we selected the minimum criteria with higher accuracy to construct the LASSO classifier, with the identification of 9 feature genes (Fig. 8A–B). This 10-fold cross-validation approach was used to minimize model overlifting and enhance the generalizability of the results. SVM-RFE classifier error was minimized when the number of features was 13 (Fig. 8C–D). The optimization was performed through interative cross-validation to ensure selection stability and prevent overfitting. The Random Forest algorithm identified 7 feature genes of relative importance by MeanDecreaseGini > 2.5 (Fig. 8E–F). After crossover, 6 feature genes (CBLB, PSMD6, RNF115, SMAD3, UCHL3 and ZBTB16) were consistently identified and shown in Figure 8G. The threshold of MeanDecreaseGini > 2.5 was determined based on the distribution of feature importance scores, which exhibited a typical long-tail pattern. The value of 2.5 was located near the “elbow point” of the distribution curve, effectively distinguishing strong predictors from noise features. This data-driven approach was used to enhance the robustness of feature selection within this study (Fig. 8H).

### 3.9. Expression validation and performance evaluation of characterized genes

**Figure 9.**
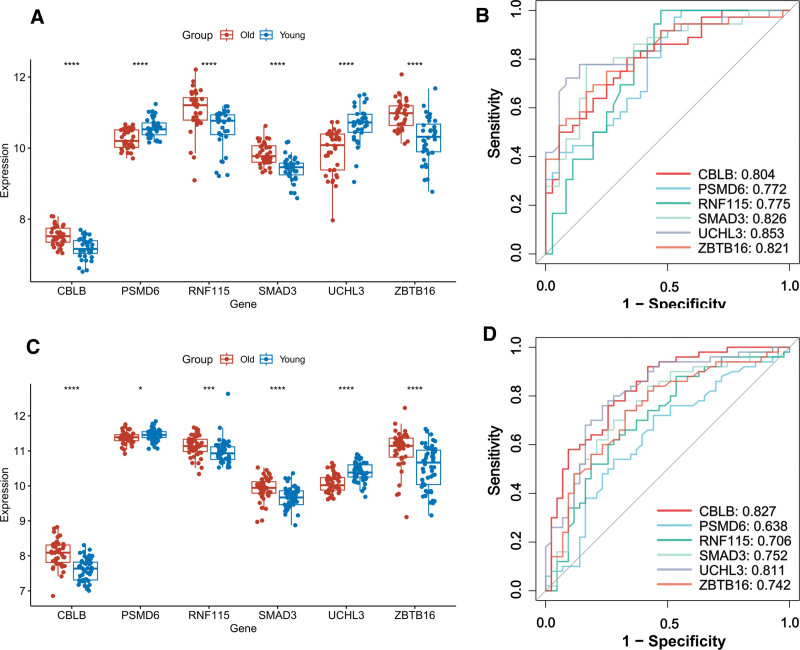
(A) and (C) Expression box plots of characterized genes. (B) and (D) Predicted ROC curves for characterized genes.

All the feature genes in the training and validation sets (Fig. 9), except for PSMD6 gene, showed significant differences between the Old and Young groups. In addition, the ROC curves further confirmed that the characterized genes had outstanding predictive value. The 5 genes of CBLB, RNF115, SMAD3, UCHL3, and ZBTB16 that passed the validation were used for subsequent analysis. To further evaluate the performance of the diagnostic models, we calculated additional indicators including sensitivity, specificity, precision, and accuracy for both training and test sets (Table 2). In the training cohort, genes such as *UCHL3*, *SMAD3*, and *CBLB* showed strong discriminative ability, with AUC values above 0.80 and overall accuracy closing to 0.80. Similar results were observed in the test cohort, where *UCHL3* (AUC = 0.811), *CBLB* (AUC = 0.827), and *SMAD3* (AUC = 0.752) maintained balanced sensitivity (approximately 0.75–0.80) and specificity (approximately 0.74–0.77). The consistent performance across datasets suggests that the model generalized well and unlikely to be affected by overfitting, confirming the diagnostic stability of the identified feature genes.

**Table 2:** Performance evaluation of feature genes based on machine learning models in training and test cohorts.

	Gene	AUC (95%CI)	Sensitivity	Specificity	Precision	Accuracy
Train	CBLB	0.804 (0.705,0.903)	0.806	0.667	0.707	0.736
	PSMD6	0.772 (0.666,0.879)	1	0.444	0.643	0.722
	RNF115	0.775 (0.664,0.885)	1	0.528	0.679	0.764
	SMAD3	0.826 (0.729,0.923)	0.778	0.833	0.824	0.806
	UCHL3	0.853 (0.763,0.942)	0.778	0.861	0.848	0.819
	ZBTB16	0.821 (0.724,0.917)	0.75	0.75	0.75	0.75
Test	CBLB	0.827 (0.743,0.912)	0.76	0.744	0.776	0.753
	PSMD6	0.638 (0.523,0.753)	0.72	0.558	0.655	0.645
	RNF115	0.706 (0.597,0.814)	0.88	0.465	0.657	0.688
	SMAD3	0.752 (0.65,0.854)	0.84	0.581	0.7	0.72
	UCHL3	0.811 (0.721,0.901)	0.76	0.767	0.792	0.763
	ZBTB16	0.742 (0.639,0.844)	0.74	0.674	0.725	0.71

### 3.10. Drug predictive analysis

**Figure 10.**
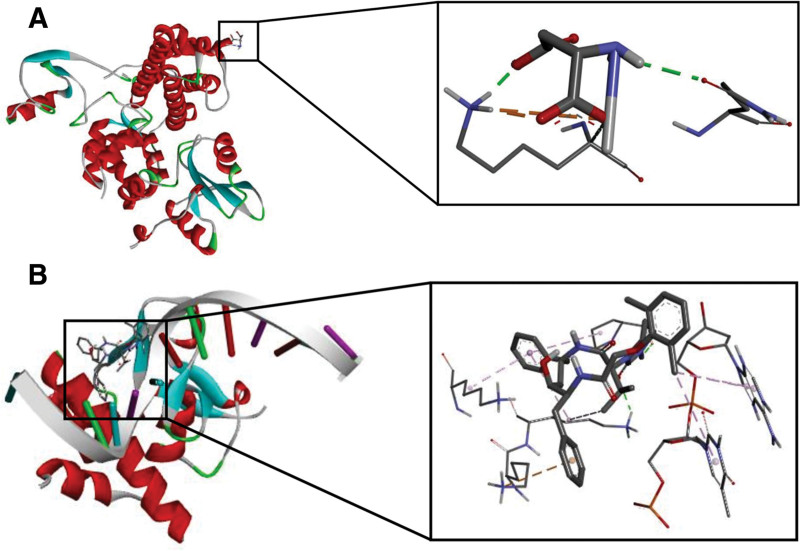
Molecular docking results. (A) 8qtk docking results, (B)1ozj docking results.

We obtained 2 of the 5 characterized genes, CBLB and SMAD3, which corresponded to the drugs ASPARTIC ACID and CETYLPYRIDINIUM, respectively, from the DGldb database. CBLB corresponded to the protein 8qtk that bound to ASPARTIC ACID with a binding energy of -2.41kcal/mol. Meanwhile, SMAD3 corresponded to the protein 1ozj that bound to CETYLPYRIDINIUM with a binding energy of -4.18kcal/mol. The molecular docking results are shown in Figure 10.

### 3.11. Molecular mechanisms of characterized genes

**Figure 11.**
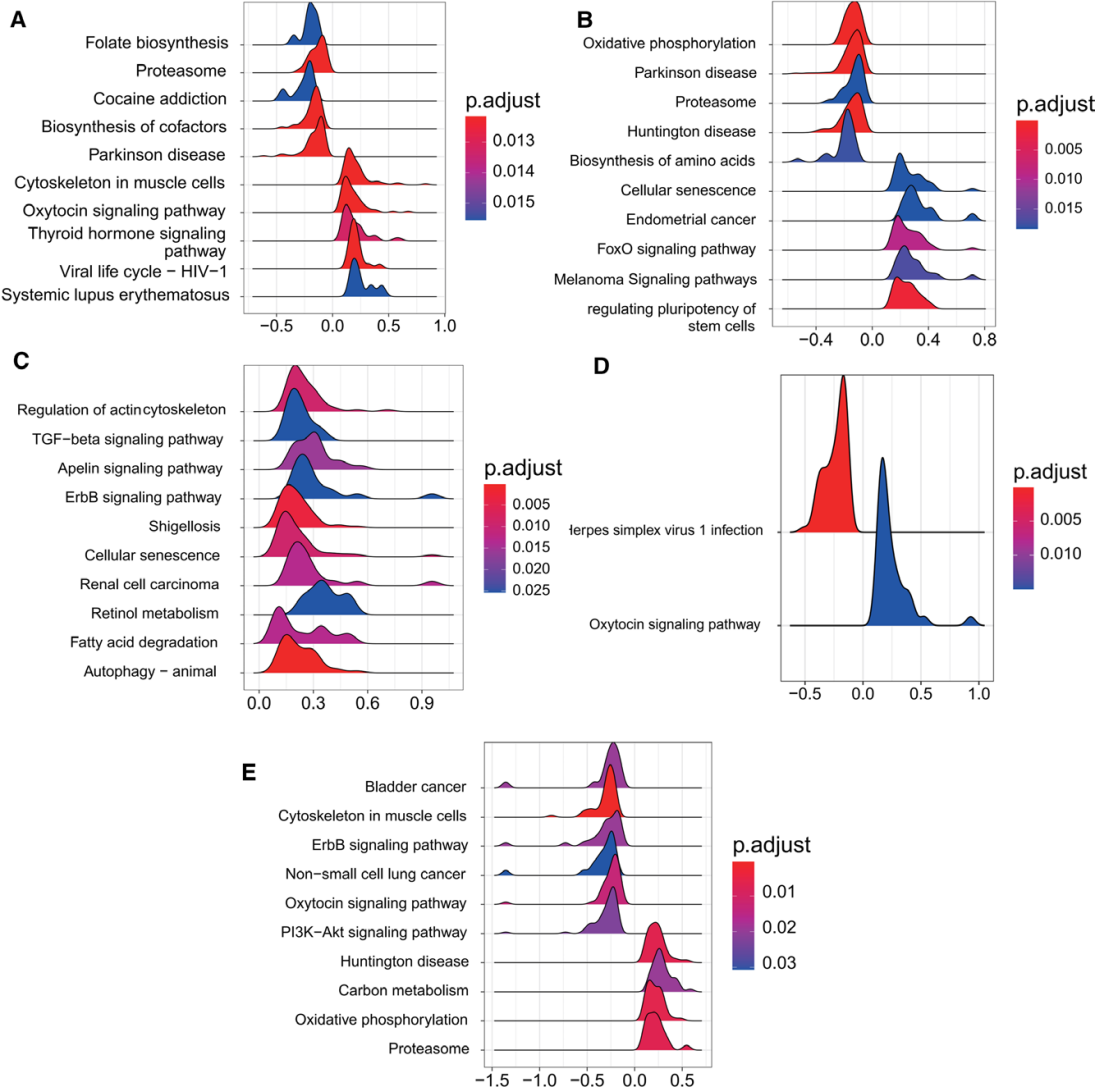
Enrichment of gene set pathways involving 5 characterized genes. (A) CBLB; (B) RNF115; (C) SMAD3; (D) UCHL3; (E) ZBTB16.

KEGG signaling pathway involved in the screened 5 characterized genes was analyzed by GSEA analysis with reference to thresholds introduced in the methodology. The results to illustrate the top10 list are shown in Figure 11.

## 4. Discussion

### 4.1. Significance of the study

In this study, we performed an integrative bioinformatics analysis to identify ubiquitination related genes that may potentially involve in the development of sarcopenia. With the increasing global aging, sarcopenia has emerged as a major threat to the health and quality of life of the elderly. In response to this urgent medical challenge, researchers worldwide are making unremitting efforts to understand its underlying molecular mechanisms and explore effective treatment methods. Here, we systematically analyzed a set of ubiquitination related genes and their functions in the UPS to provide new insights into the molecular pathways contributing to sarcopenia. This study highlights the central role of UPS in the pathological process of sarcopenia and suggested that precise regulation of ubiquitination pathways could offer promising directions for the treatment of sarcopenia, bringing new hope for improving the quality of life of elderly patients.

Although sarcopenia involves multiple biological pathways such as mitochondria dysfunction, defective autophagy, oxidative stress and inflammatory signaling, UPS was selected as key focus of this study because its fundamental role in protein homeostasis. UPS functions as the main proteolytic mechanism for degradation of myofibrillar proteins, which are essential for maintaining muscle structure and contractile activity.^[7,29]^ Dysregulation of UPS has been reported linking to enhanced proteolysis and muscle atrophy during aging.^[30]^ In contrast, other pathways such as mitochondria quality control and autophagy also influence muscle health. These systems work in coordination with UPS, supporting energy balance, removing damaged organelles and preserving overall cellular integrity.^[31–33]^ By focusing on UPS, this study offers a direct molecular perspective on the proteolytic imbalance underlying sarcopenia while recognizing that integration of these complementary pathways in future research will provide a better understanding of disease progression.

### 4.2. Validation of key genes

By using ML algorithms and expression analysis techniques, this study further validated several URGs including CBLB, PSMD6, RNF115, SMAD3, UCHL3 and ZBTB16 as potential markers associated with sarcopenia. These genes showed significantly different expression levels between the Old and Young groups, and their diagnostic performance was supported by operating characteristic (ROC) curve analysis. The additional evaluation of sensitivity, specificity, precision and accuracy further confirmed their predictive reliability. These findings enhance the robustness of our bioinformatic results and suggest the potential of these genes as diagnostic biomarkers and therapeutic targets for sarcopenia.

Notably, several of these genes have previously been linked to muscle or proteostasis regulation. Among these, *CBLB* encodes an E3 ubiquitin ligase that negatively regulates IGF-1/PI3K/Akt signaling and contribute to skeletal muscle atrophy by promoting the degradation of insulin receptor substrate-1 (IRS-1) and downstream anabolic signaling components.^[34,35]^ *SMAD3* is a downstream mediator of TGF-β signaling and has been shown to regulate satellite cell differentiation and myogenic commitment. Inhibition or loss *SMAD3* activity results in impaired myogenesis and abnormal muscle regeneration.^[36]^ Therefore, dysregulated TGF-β/SMAD3 signaling is considered a major regulator to age related muscle fibrosis and atrophy.^[37]^ *UCHL3* is a de-ubiquitinating enzyme which plays an important role in maintaining skeletal muscle proteostasis. Previous study had shown that *Uchl3*-deficient mice exhibit pronounced accumulation of polyubiquitinated proteins and activation of stress markers such as Grp78, PDI and heat shock proteins, indicating that loss of UCHL3 disrupts ubiquitin turnover and induces cellular stress in muscle tissue.^[38]^

Although *ZBTB16* and *RNF15* are not yet directly linked to sarcopenia, current evidence suggests that they participate in biological pathways relevant to muscle metabolism and proteostasis. *ZBTB16* has been associated with glucocorticoid-mediated metabolic regulation. In congenic rat models, presence of a variant *Zbtb16* locus led to significant reduction in insulin-stimulated glucose incorporation into skeletal muscle glycogen following dexamethasone treatment, suggesting a potential role in muscle energy homeostasis.^[39]^ RNF115, an E3 ubiquitin ligase localized to endosomal and phagosomal membranes, is known to regulate ubiquitin dependent intracellular trafficking and innate immune responses.^[40]^ Although its function in skeletal muscle has not been investigated, these processes may intersect with inflammatory and proteolytic mechanisms that contribute to muscle atrophy. Together these findings suggest that *ZBTB16* and *RNF15* may represent previously unrecognized regulators in sarcopenia and should be further investigated.

### 4.3. Drug predictive analysis

In this part, CBLB was found to be associated with aspartic acid, while SMAD3 was related to cetylpyridinium. Through molecular docking, these drugs had good binding affinity with the targets, suggesting their potential in the treatment of sarcopenia. This discovery may offer valuable reference for drug development and drug repurposing, potentially accelerating the discovery of effective strategies for sarcopenia. Moreover, identification of existing drugs that can interact with these genes may imply the potential for rapid clinical translation.

Although the calculated binding energies (e.g., −2.41 kcal/mol) indicating relatively weak affinities, these results primarily serve as preliminary computational evidence of possible drug targeted interactions rather than definitive proof of biological activity. Therefore, these findings should be regarded as exploratory and will need to be validated through further biochemical and cellular experiments to confirm their therapeutic significance in sarcopenia.

### 4.4. Molecular mechanisms of characterized genes

Through GSEA of the identified genes, we may acquire useful data to get into the bottom of their roles in various signaling pathways. These genes were significantly enriched in specific KEGG pathways, suggesting their profound roles in regulating muscle protein degradation, inflammation, and other biological processes related to sarcopenia. Further studies of these pathways may reveal more detailed mechanisms and potential targets for intervention. In addition, deciphering the participation of these genes in sarcopenia may advance the development of targeted therapies.

### 4.5. Limitations of the study

Despite the aforementioned encouraging results, this study still possesses several deficiencies. First, our implementation of public data-based bioinformatics analysis might produce smaller sample size and insufficient population representation. In addition, the modest sample sizes of the individual datasets may limit statistical power, particularly for small effect sizes. However, integrating multiple cohorts and applying stringent analytical methods would help improve robustness. Future studies should include larger and more diverse sample groups to validate the findings. Second, our study did not include experimental validation (e.g., qPCR, Western blotting, or functional assays) to further confirm the bioinformatics predictions and clarify the interactions of the screened genes with the predicted drugs. We acknowledge this as a major limitation and the future studies should be performed, including gene expression confirmation in independent cohorts, protein-level verification through Western blotting, and functional assays to assess the biological roles of key genes. Third, our study emphasized on UPS merely, without further consideration of other possible pathways and mechanisms in the development of sarcopenia. In the future, a more comprehensive analysis involving multiple pathways and systems may provide a more holistic understanding of the disease.

### 4.6. Future directions

In our future research, it is feasible to conduct n vitro and in vivo experiments for validating the identified genes and their interactions with the predicted drugs. It may benefit the confirmation of their therapeutic potential and lay the foundation for clinical trials. It may be promising to explore the interactions between the identified genes and other biological pathways, thereby revealing more complex mechanisms underlying sarcopenia. In addition, another direction may include the development of personalized treatment strategies based on individual genetic profiles, thus offering more effective and targeted therapies for sarcopenia. Collaboration among bioinformaticians, molecular biologists, and clinicians may also be a crucial direction for translating these research findings into clinical practice.

## 5. Conclusion

In summary, this study identifies multiple URGs that have potential therapeutic implications for sarcopenia, facilitating further research and exploition of new therapies for this debilitating condition. Validation of these genes highlights their potential as diagnostic markers and therapeutic targets. Despite the limitations of the current study, these findings provide highly promising directions for future research and clinical applications. To deepen our understanding and treatment of sarcopenia, it is still important to perform further experimental validation and exploration of the molecular mechanisms behind these genes.

## Author contributions

**Conceptualization:** XiaoMing Liu.

**Data curation:** XiaoMing Liu, Ren Li.

**Validation:** YingYan Kuang.

**Visualization:** XiaoMing Liu, Ren Li.

**Writing – original draft:** XiaoMing Liu, Ren Li, YingYan Kuang.
